# The impact of esophagogastric varices on the prognosis of patients with hepatocellular carcinoma

**DOI:** 10.1038/srep42577

**Published:** 2017-02-17

**Authors:** Wei-Yao Hsieh, Ping-Hsien Chen, I-Yen Lin, Chien-Wei Su, Yee- Chao, Teh-Ia Huo, Yi-Hsiang Huang, Ming-Chih Hou, Han-Chieh Lin, Jaw-Ching Wu

**Affiliations:** 1 Division of Gastroenterology and Hepatology, Department of Medicine, Taipei Veterans General Hospital, Taipei, Taiwan; 2 Faculty of Medicine, School of Medicine, National Yang-Ming University, Taipei, Taiwan; 3 Endoscopy Center for Diagnosis and Treatment, Taipei Veterans General Hospital, Taipei, Taiwan; 4 Institute of Biophotonics, National Yang-Ming University, Taipei, Taiwan; 5 Department of Nursing, Taipei Veterans General Hospital, Taipei, Taiwan; 6 Department of Oncology, Taipei Veterans General Hospital, Taipei, Taiwan; 7 Department and Institute of Pharmacology, School of Medicine, National Yang-Ming University, Taipei, Taiwan; 8 Institute of Clinical Medicine, School of Medicine, National Yang-Ming University, Taipei, Taiwan; 9 Division of Translational Research, Department of Medical Research, Taipei Veterans General Hospital, Taipei, Taiwan

## Abstract

Whether or not esophagogastric varices (EGV) could determine the outcomes of patients with hepatocellular carcinoma (HCC) is still unclear. A total of 990 treatment-naive HCC patients who received an esophagogastroduodenoscopy at the time of HCC diagnosis were retrospectively enrolled. The factors in terms of prognosis were analyzed by Cox proportional hazards model and propensity score matching analysis. Among the enrolled patients, 480 (48.5%) patients had EGV. Patients with EGV had a significantly lower cumulative 5-year survival rate than those without EGV (24.9% versus 46.4%, *p* < 0.001). It was confirmed by a multivariate analysis and propensity score matching analysis. Stratified by tumor stage, the patients with EGV had lower survival rates than the patients without EGVs in all Barcelona Clinic Liver Cancer stages except stage D. Moreover, the patients with EGV had lower survival rates than those without EGV, both by curative or non-curative treatment modalities. In conclusion, EGV was an independent risk factor predicting poor prognosis for the patients with HCC by multivariate analysis, propensity score matching analysis, and subgroup analysis.

Hepatocellular carcinoma (HCC) is one of the leading causes of cancer mortality worldwide^1^,^2^. With the improvement in the surveillance, diagnosis, and treatment of HCC, the outcomes of patients with HCC have been improved substantially, but these outcomes are still unsatisfactory^3^,^4^,^5^,^6^. One recent survey from the United States demonstrated that the 5-year overall survival (OS) rates for patients with liver cancer increased from 3% between 1975 and 1977 to 18% between 2004 and 2010^2^. To improve the prognosis of patients with HCC, it is crucial to elucidate the mechanism of hepatocarcinogenesis and identify the prognostic factors. Factors affecting the outcomes of HCC include host factors (such as age, gender, genetic factors, and performance status), tumor factors (including tumor burden, the presence of vascular invasion or extra-hepatic metastasis, and tumor cell differentiation), liver functional reserve factors (Child–Pugh classification, portal hypertension [PHT], and platelet count), and treatment modality factors^7^,^8^,^9^,^10^,^11^.

Notably, more than 80% of patients with HCC have concomitant liver cirrhosis^12^,^13^. The simultaneous presence of two distinct diseases makes the prognostic assessment and the treatment strategy very complex in these patients. Among the cirrhotic patients, esophagogastric varices (EGV) are one of the major complications of PHT and are responsible for subsequent mortality^14^,^15^,^16^,^17^. Several previous studies further demonstrate that more than 50% of patients have concomitant EGV when HCC is diagnosed^18^,^19^. However, whether or not EGV are associated with the prognoses of patients with HCC is not yet fully elucidated^18^,^20^,^21^. We deduced that HCC patients with EGV might have relatively poor liver functional reserves, leading to poor outcomes. To validate this hypothesis, this study aimed to investigate the impact of EGV on the clinical manifestations, treatment modalities, and outcomes for patients with HCC.

## Results

### Baseline clinical characteristics

**Figure 1:**
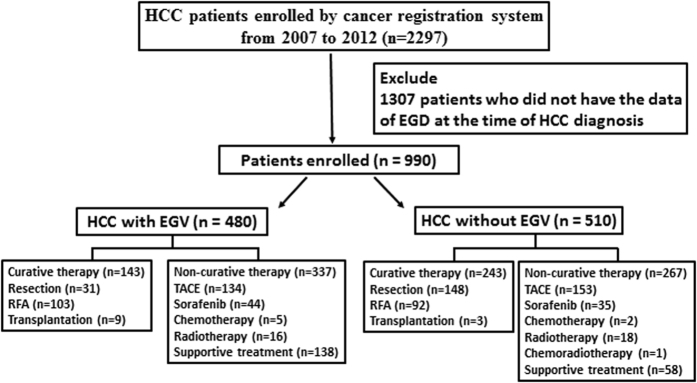
The study flow chart.

The study flow chart is depicted in Fig. 1 and the main demographic and clinical data of the study population are shown in Table 1. Among the 990 patients who received an esophagogastroduodenoscopy (EGD), 942 (95.2%) patients were due to screening for EGV and the remaining 48 (4.8%) patients were due to bleeding, including 15 patients with EGV bleeding and 33 patients with peptic ulcer bleeding, respectively. Among the 480 (48.5%) patients who had EGV diagnosed by an EGD, 399 patients had esophageal varices (EV) alone, and 12 patients had gastric varices (GV) alone. The remaining 69 patients had both EV and GV. For the 468 patients with EV, the varix size was small in 203 patients (43.4%), medium in 192 patients (41.0%), and large in 73 patients (15.6%).

Compared to those without EGV, the patients with EGV were younger in age and had lower platelet counts; higher serum alanine aminotransferase (ALT), aspartate aminotransferase (AST), and alkaline phosphatase (Alk-P) levels; higher rates of hepatic encephalopathy; higher rates of ascites; more prolonged prothrombin times; lower serum albumin levels; higher serum bilirubin; lower rates of Child-Pugh grade A; and higher scores of model for end-stage liver disease (MELD). Regarding tumor factors, the patients with EGV had smaller tumor sizes, but they had more multinodular lesions, more rates of tumor vascular invasion, more advanced tumor stages, and lower rates for receiving curative treatments.

### Factors associated with poor OS rates

**Figure 2:**
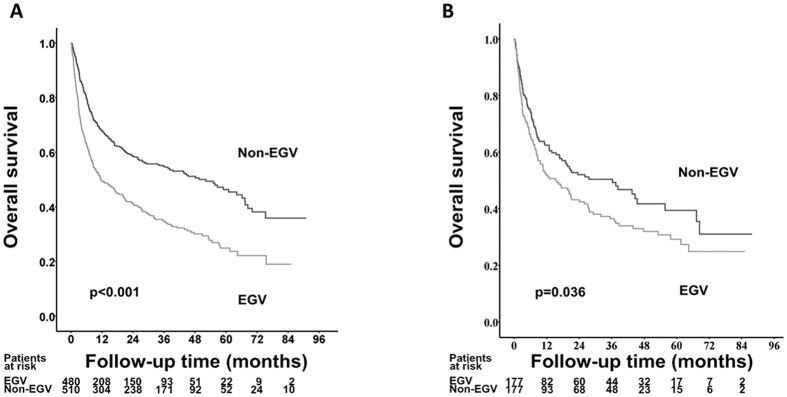
Comparison of cumulative overall survival rates between EGV and non-EGV cohorts before (**A**) and after (**B**) propensity score matching analysis.

After a median follow-up of 13.1 months (interquartile range, 3.6–36.9 months), 533 patients died, and the remaining 457 patients were still alive at their last visit. As shown in Fig. 2A, the cumulative 1-, 2-, 3-, and 5-year OS rates were 49.4% vs. 67.7%, 41.1% vs. 58.6%, 34.7% vs. 54.9%, and 24.9% vs. 46.4% in the patients with and without EGV, respectively (*p* < 0.001).

A multivariate analysis disclosed that male sex, serum albumin ≤4.0 g/dL, bilirubin >1.6 mg/dL, and AST > 45 U/L, along with indicators of PHT (such as the presence of EGV and a platelet count ≤100,000/mm^3^), were the significant predictors of poor OS rates (Table 2). Among the tumor-related factors, a serum alpha-fetoprotein (AFP) level >20 ng/mL, multiple tumors, a tumor size >3 cm, the presence of vascular invasion, and non-curative treatments were also the independent risk factors associated with poor OS rates.

As the demographic and tumor characteristics were diverse between patients with and those without EGV, a propensity score matching analysis was further performed to minimize the confounding factors that might determine the prognosis. And 177 patients were matched in each group by one-to-one nearest-neighbor matching method. After matching, the demographic characteristics, tumor factors, and treatment modalities were well-matched between the two groups (Supplementary Table S1). After matching, HCC patients with EVG still had a lower OS rates than those without EGV (Fig. 2B, *p* = 0.036).

### The impact of EGV on OS rates for HCC patients by different tumor stages and subgroup analysis

**Figure 3:**
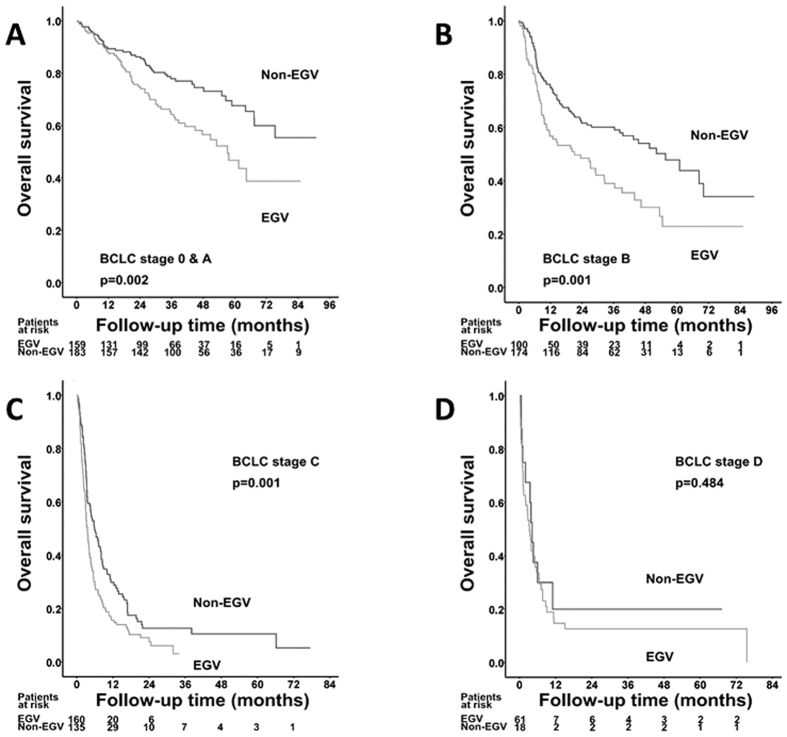
Comparison of overall survival rates between EGV and non-EGV cohorts stratified by BCLC stages (**A**: stage **A**; **B**: stage **B**; **C**: stage **C**; and **D**: stage **D**).

**Figure 4:**
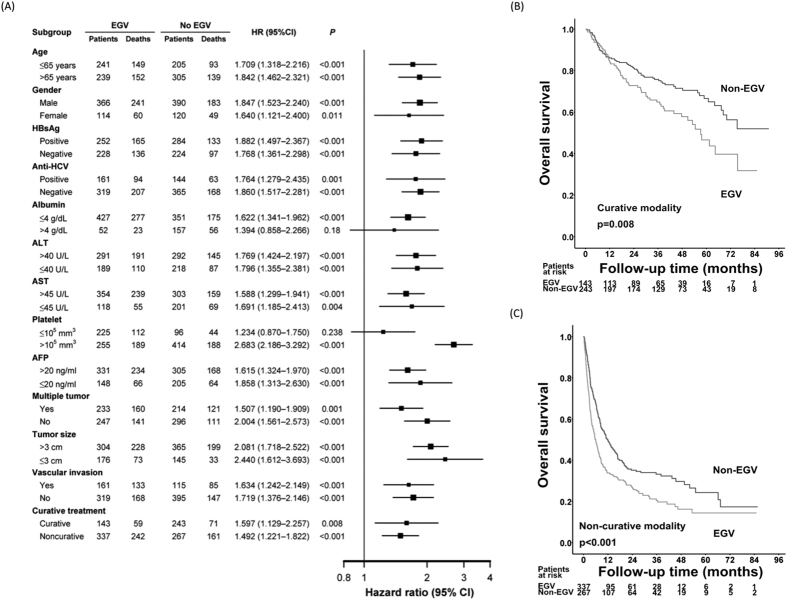
Comparison of overall survival rates between EGV and non-EGV cohorts stratified by different demographic characteristics (**A**) and treatment modalities (**B**: curative therapy; **C**: non-curative therapy).

When stratified by Barcelona Clinic Liver Cancer (BCLC) stage, the patients with EGV had lower OS rates than those without EGV in all of the BCLC stages except stage D (Fig. 3A–D). A subgroup analysis further demonstrated that the HCC patients with EGV had significantly poorer OS rates than those without EGV in most of the subgroups except in regards to serum albumin > 4 g/dL and a platelet count ≤ 10^5^/mm^3^ (Fig. 4A).

### The impact of EGV on OS rates for HCC patients undergoing curative therapies

Among the 386 patients who underwent curative therapies, 143 patients had EGV, and 243 patients did not have EGV at the time of HCC diagnosis. The cumulative 1-, 2-, 3-, and 5-year OS rates were 84.7% vs. 86.1%, 72.8% vs. 81.1%, 64.8% vs. 75.7%, and 46.6% vs. 66.5% in the patients with and without EGV, respectively (Fig. 4B). The patients with EGV had significantly lower survival rates than those without EGV (*p* = 0.008).

Multivariate analyses showed that a platelet count ≤ 100,000/mm^3^, a serum AFP level >20 ng/mL, a tumor size > 3 cm, the presence of vascular invasion, and the presence of EGV were the independent risk factors that predicted poor OS rates after curative therapies (Table 3).

### The impact of EGV on OS rates for HCC patients undergoing non-curative therapies

For those who received non-curative treatments, the cumulative 1-, 2-, 3-, and 5-year OS rates were 33.8% vs. 49.3%, 26.6% vs. 35.1%, 20.5% vs. 33.2%, and 14.4% vs. 24.3% in the patients with and without EGV, respectively (Fig. 4C). The patients with EGV had significantly lower survival rates than those without EGV (*p* < 0.001).

A multivariate analysis disclosed that male gender, serum albumin ≤4.0 g/dL, bilirubin >1.6 mg/dL, a serum AFP level >20 ng/mL, a tumor size >3 cm, the presence of vascular invasion, as well as the presence of EGV, were the independent risk factors associated with poor OS rates (Table 4).

### The impact of primary prophylaxis for variceal bleeding on the prognoses of HCC patients with medium and large varices

**Figure 5:**
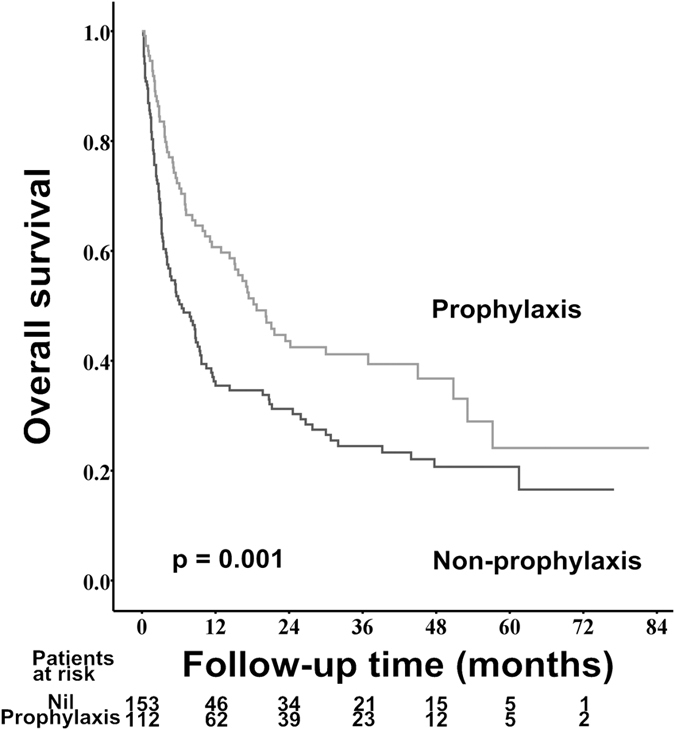
Comparison of overall survival rates between primary prophylaxis and non-prophylaxis cohorts in patients with medium and large esophageal varices.

Among the 265 patients who had medium and large esophageal varices, 112 (42.3%) patients received primary prophylaxis for variceal bleeding, including 82 patients received endoscopic band ligation (EBL) and the remaining 30 patients took nonselective beta-blockers (NSBB). The cumulative 1-, 3-, and 5-year OS rates were 60.7% vs. 35.5%, 41.2% vs. 24.5%, and 24.1% vs. 20.7% for patients with and without primary prophylaxis for variceal bleeding, respectively (*p* = 0.001, Fig. 5).

## Discussion

There are several major findings of this study. First, EGV were common in the HCC patients; EGV were found in nearly half of the patients in our cohort study. Second, the HCC patients with EGV had significantly poorer liver functional reserves and more advanced tumor stages as compared with those patients without EGV at the time of HCC diagnosis. Third, patients with EGV had significantly lower OS rates than their counterparts. Of note, the trend was consistent in most of the subgroup analyses stratified by different demographic characteristics, HCC tumor stages, as well as treatment modalities, and it was further confirmed by a multivariate analysis and propensity score matching analysis. The patients with EGV have a 32.4% increased risk of death as compared with the patients without EGV, suggesting that EGV is an important prognostic factor for HCC.

For the patients with chronic hepatitis, PHT, which is defined as a hepatic venous pressure gradient (HVPG) greater than 5 mmHg, occurs frequently with the progression of liver fibrosis^17^. Previous studies demonstrated that when the HVPG is greater than 10 mmHg, the incidence of EGV development, the emergence of liver decompensation and HCC would increase substantially^16^,^22^,^23^. Hence, an HVPG greater than 10 mmHg has been designated as clinically significant portal hypertension (CSPH)^16^. However, the measurement of the HVPG is invasive, costly, and infeasible for most hospitals. Therefore, indirect clinical parameters, such as the presence of EGV and/or splenomegaly in association with a platelet count of less than 100,000/mm^3^, are considered as the surrogates of CSPH to indicate the presence and degree of PHT^24^. Nonetheless, previous studies showed that in patients who have no EGV, platelet count and spleen size are not accurate enough to rule out the diagnosis of CSPH in up to 40% of the cases^25^,^26^. On the other hand, as a consequence of PHT, it has been demonstrated that the threshold of the HVPG for the development of EV is above 10 mmHg^27^,^28^. Hence, the formation of EGV might be sufficient to confirm the presence of CSPH.

In this study, compared to the HCC patients without EGV, those patients with EGV had lower platelet counts; lower serum albumin levels; higher ALT, AST, and bilirubin levels; and higher Child-Pugh scores, indicating that they had poorer liver functional reserves, more active hepatic necro-inflammation, and even advanced fibrosis. These findings are consistent with the previous reports^18^,^20^,^21^,^29^. As these factors are critical in determining the prognosis of the patients with HCC, CSPH and EGV have been incorporated into the BCLC staging system and are widely applied as important references when selecting the treatment modalities in daily practice^24^,^30^.

The current guidelines for the management of HCC recommend resection surgery, liver transplantation, and local ablation therapy as the curative treatment modalities for the very early and early stages (BCLC 0–A) of HCC^24^,^31^. However, the indication of resection surgery has been limited to those patients without CSPH^30^. It is based on the negative impact of PHT and CSPH on the postoperative prognoses, including post-hepatectomy liver failure and long-term survival, demonstrated by several prospective studies and meta-analyses^9^,^29^,^32^,^33^. However, several recent studies demonstrate that CSPH is not a contraindication of resection for HCC^20^,^21^,^34^,^35^,^36^. Harada disclosed that HCC patients with EV could achieve similar long-term outcomes as those patients without EV if an indocyanine green retention test at 15 minutes (ICGR15) ≤17%^20^. Another recent study conducted by Cucchetti further showed that, although measuring the HVPG could be used for predicting the risk of hepatic decompensation after resection surgery for HCC, the current recommended threshold of 10 mmHg in determining CSPH might too low, which in turn excluded around one-quarter of the patients suitable for resection surgery^29^. Consequently, the role of CSPH, EGV, and ICGR15 in stratifying the indication and the extent of a hepatectomy continues to be debated and needs further studies to elucidate this issue. Moreover, the impact of CSPH on the outcomes of HCC patients who undergo treatments other than surgical resection remains unclear as well. In our current study, the patients with EGV had poorer outcomes than their counterparts, and the results were consistent across different BCLC tumor stages except for the terminal stage and most of the subgroup analysis. Moreover, EGV was associated with lower OS rates both in patients receiving curative and non-curative therapies for HCC. These findings validated that EGV are a poor prognostic factor for HCC due to more advanced liver fibrosis and a poor liver functional reserve.

In our cohort study, the prevalence of EGV in patients with HCC was 48.5%, which was lower than that observed in the Italian Liver Cancer Group cohort study (63.3%)^18^. This difference might be due to the discrepancy of the HCC etiology between the Eastern and Western countries. In Taiwan, the predominant cause of HCC is the hepatitis B virus (HBV) infection, which could cause hepatic carcinogenesis in the absence of advanced hepatic fibrosis or cirrhosis^8^,^37^. By contrast, the major etiology of HCC in the Western countries is chronic hepatitis C virus (HCV) infection, and the majority of the patients developed HCC after long-term chronic inflammation, fibrosis, and cirrhosis. Consequently, it is reasonable that patients in the Eastern countries have lower rates of EGV at the time of HCC diagnosis due to less cases of liver cirrhosis. However, although the etiology of HCC and the prevalence of EGV are diverse between patients in Eastern and Western countries, both of these studies confirmed that EGV were associated with poor prognoses of patients with HCC, irrespective of viral etiologies, tumor stages, and treatment modalities. Clinical physicians should take EGV into account when adopting the strategy of therapy and predicting outcomes for patients with HCC.

Notably, the rate of performing EGV by an EGD in our cohort study was only 43.1%, which was relatively lower compared to previous reports (50–63%) in Western countries^18^,^38^. Besides the different viral etiologies of HCC between Eastern and Western countries, the unawareness of compensated liver cirrhosis due to the lack of clinical symptoms might be a more important factor to explain the lower rates of screening EGDs at the time of HCC diagnoses. One recent study showed that cirrhosis was unrecognized prior to HCC diagnoses in approximately 24.6% of patients^13^. However, the presence of EGV actually affects the selection of treatment modalities and prognoses. Consequently, it is recommended to arrange an EGD to screen EGV for HCC patients, especially for those with underlying liver cirrhosis.

According to the current Baveno VI consensus for the management of portal hypertension, either NSBB or EBL is recommended to prevent the first variceal bleeding in cirrhotic patients with medium or large varix^16^. Nevertheless, the role of primary prevention for EV bleeding in HCC patients is still obscure and only several studies with limited number of patients focus on this issue till now^39^,^40^. In our current study, only 42.3% of patients who bore a high risk of EV bleeding received primary prophylaxis therapy. It might reflect the lack of awareness for the risk of EV bleeding in HCC patients by some clinical physicians in the real world practice. Of note, our large cohort study demonstrated the survival beneficial effect of primary prevention for EV in patient with HCC. But this result might be limited by the retrospective study design. Further prospective studies investigating the role of primary prevention for EV bleeding in HCC patients are warranted.

We acknowledge some limitations of our study. First, not every patient in the registration database received an EGD for EGV assessment when HCC was diagnosed. And it was difficult to compare the demographic characteristics, tumor factors, and the status of EGV, between patients with and without receiving EGD at the time of HCC diagnosis. Selection bias might be present because of the retrospective nature of this study. For instance, an EGD might be arranged according to a physician’s concern and a patient’s consent. In addition, an EGD might be performed on a patient who had a bleeding episode; however, an EGD might not be arranged for a patient with a poor clinical condition. Consequently, it could not accurately estimate the prevalence rate of EGV in HCC patients. However, the aim of this study was to assess the impact of EGV on the prognoses of patients with HCC. Our results demonstrated that EGV was an independent risk factor associated with poor prognosis in HCC patients confirmed by a multivariate analysis, propensity score matching analysis, and subgroup analysis across different viral etiologies, demographic characteristics, liver functional reserve, and treatment modalities. It could provide a more robust evidence to remind the clinical physicians to arrange an EGD while diagnosing a patient with HCC. Second, the documentation of EGV status was at the beginning of this study only. Data regarding those patients who developed EGV during the follow-up period were unavailable. Third, we analyzed the prognostic weight of EGV without subdividing the patients according to the characteristics of EGV, such as the size and the presence of red color signs, the natural history of EGV (such as the progression in variceal size and variceal bleeding episodes), as well as the different treatments and prophylaxis strategies toward variceal bleeding, which are suggested by international guidelines. These confounding factors, including the relatively poorer liver functional reserve in patients with EGV, the poorer performance status and clinical condition in patients with bleeding as the presentation, and lower rate of primary prophylaxis for high risk EGV in our cohort, might affect the impact of EGV on determining the outcomes of HCC patients. In spite of these limitations, our large-scale study composed of 990 HCC patients, including detailed demographic, endoscopic, and tumor data and a comprehensive analysis of prognostic factors, could provide robust evidence to elucidate the role of EGV in determining the outcomes of patients with HCC. But further prospective studies are warranted to fully elucidate this issue.

## Conclusions

The HCC patients with EGV had relatively poorer liver functional reserves than those without EGV. Moreover, EGV were an independent risk factor predicting OS rates by multivariate analysis, propensity score matching analysis, and subgroup analysis.

## Methods

### Patients and follow-up

This cohort study was prospectively conducted and retrospectively analyzed. The database contained 2297 consecutive treatment-naive patients who fulfilled the diagnostic criteria of HCC by the American Association for the Study of Liver Disease and were enrolled in the cancer registration system from October 2007 to October 2012 at Taipei Veterans General Hospital^24^. All of the HCC patients were discussed in terms of diagnoses and treatment strategies at a weekly multidisciplinary meeting. They underwent thorough clinical, laboratory, and image assessments and were followed up every 3 months until their last visit to the hospital, their death, or October 31, 2012. After excluding 1307 patients who did not receive an EGD at the time of HCC diagnosis, a total of 990 patients were recruited for the final analysis.

After the physicians explained the advantages, side effects, and prognoses of various therapy modalities and the recommendations from the multidisciplinary experts, the number of patients undergoing curative treatments were as follows: liver transplantation for 12 patients, resection surgery for 179 patients, and radiofrequency ablation (RFA) for 195 patients. The non-curative treatments included transarterial chemoembolization (TACE) and others (e.g., sorafenib, supportive care alone, chemotherapy, radiotherapy, or chemo-radiotherapy), which enrolled 287 and 317 patients, respectively. The study was conducted in accordance with the Declaration of Helsinki and current ethical guidelines. It was approved by the Institutional Review Board (IRB) of the Taipei Veterans General Hospital. As a retrospective cohort data, informed consent was waived by IRB. Patient information was de-identified before the initiation of this study.

### Biochemical and serologic markers

Serum hepatitis B surface antigen (HBsAg) and the HCV antibody were tested by radio-immunoassay (Abbott Laboratories, North Chicago, IL) and second-generation enzyme immunoassay (Abbott). Serum biochemistries were measured using a Roche/Hitachi Modular Analytics System (Roche Diagnostics GmbH, Mannheim, Germany). The serum AFP level was tested using a radio-immunoassay kit (Serono Diagnostic SA, Coinsin/VD, Switzerland).

### Statistical analysis

The primary endpoint was OS. This was calculated from the diagnosis of HCC to the patient’s death, the patient’s last visit, or the loss of the patient during follow-up^30^. A Fisher’s exact test or a chi-squared test with Yates’ correction was performed to compare the categorical variables when appropriate, and the Mann–Whitney U-test was applied to compare the continuous variables. The cumulative OS rates were estimated using the Kaplan–Meier method and compared using the Cox’s proportional hazards model. In addition, we confirmed the assumption of proportional hazards by the log-minus-log plot of survival in a Cox regression analysis.

To further minimize the potential confounding factors that might affect the prognosis, a propensity score matching analysis was performed as previously described^41^,^42^. Variables entered into the propensity model were age, serum albumin, bilirubin, ALT, AST, Alk-P, glucose levels, prothrombin times international ratio (PT INR), hemoglobin and platelet counts, status of ascites and hepatic encephalopathy, tumor size, tumor number, and vascular invasion. Subsequently, a one-to-one match between the EGV and non-EGV groups was obtained by using the nearest-neighbor matching method. Prognoses analysis was performed again to analyze the OS amended from these confounding factors.

The variables with statistical significance (*p* < 0.05) or approximate significance (*p* < 0.1) by a univariate analysis were subjected to a multivariate analysis using a forward stepwise logistic regression model. A two-tailed value of *p* < 0.05 was considered statistically significant. All statistical analyses were performed using IBM SPSS Statistics for Windows, version 21.0 (IBM Corp., Armonk, NY, USA).

## Additional Information

**How to cite this article**: Hsieh, W.-Y. *et al*. The impact of esophagogastric varices on the prognosis of patients with hepatocellular carcinoma. *Sci. Rep.* **7**, 42577; doi: 10.1038/srep42577 (2017).

**Publisher's note:** Springer Nature remains neutral with regard to jurisdictional claims in published maps and institutional affiliations.

## Supplementary Material

Supplementary Table S1

## Figures and Tables

**Table 1:** Demographic data of HCC patients with and without EGV.

Parameter	All patients (n = 990)	EGV group (n = 480)	Non-EGV group (n = 510)	*P*
**Patient Demographics**				
Age (years)	67.0; 58.0–79.0	65.0; 57.0–76.0	70.0; 60.0–81.0	0.001
Sex (male) (%)	756 (76.4%)	366 (76.3%)	390 (76.5%)	0.995
HBsAg positive/negative (%)	536/452 (54.3%/45.7%)	252/228 (52.5%/47.5%)	284/224 (55.9%/44.1%)	0.312
Anti-HCV positive/negative (%)	305/684 (30.8%/69.2%)	161/319 (33.5%/66.5%)	144/365 (28.3%/71.7%)	0.086
MELD score	8.93;7.50–11.58	10.21;8.28–13.67	7.98;7.08–9.88	<0.001
**Serum biochemistry tests**				
Albumin (g/dL)	3.50; 3.10–4.00	3.30; 2.90–3.70	3.70; 3.30–4.10	<0.001
Total bilirubin (mg/dL)	0.93; 0.61–1.58	1.25; 0.80–2.17	0.70; 0.50–1.07	<0.001
ALT (U/L)	47.0; 30.8–76.0	49.0; 33.0–83.0	45.0; 28.0–71.0	0.015
AST (U/L)	64.0; 38.0–106.0	74.0; 45.3–125.8	55.5; 35.0–89.0	<0.001
Alk-P (U/L)	111.0; 81.0–161.0	122.0; 94.0–181.0	101.0; 75.0–145.3	<0.001
Cholesterol (U/L)	154.0; 127.0–180.3	149.5; 122.8–175.8	159.0; 132.3–182.8	0.252
Creatinine (mg/dL)	0.91; 0.74–1.13	0.89; 0.71–1.13	0.93; 0.79–1.13	0.577
Glucose (mg/dL)	100.0; 85.0–129.0	105.5; 84.0–137.0	97.0; 85.0–122.0	0.049
PT INR	1.10; 1.04–1.21	1.17; 1.09–1.28	1.06; 1.00–1.13	<0.001
Platelet (/mm^3^)	128000; 88000–191000	106000; 71000–138000	166000; 111750–226250	<0.001
Ascites (yes) (%)	307 (31.0%)	232 (48.3%)	75 (14.7%)	<0.001
Hepatic encephalopathy (yes/no)	21/776 (2.6%/97.4%)	17/375 (4.3%/95.7%)	4/401 (1.0%/99.0%)	0.006
Hemoglobin (g/dL)	12.2; 10.4–13.6	11.8; 10.0–13.2	12.5; 10.8–14.0	<0.001
Child-Pugh grade (A/B/C) (%)	661/267/62 (66.8%/26.9%/6.3%)	238/191/51 (49.6%/39.8%/10.6%)	423/76/11 (82.9%/14.9%/2.2%)	<0.001
**Tumor factors**				
Tumor size (cm)	5.20; 2.60–9.60	4.60; 2.50–9.18	5.95; 2.90–9.93	0.009
Single tumor (%)	543 (54.8%)	247 (51.5%)	296 (58.0%)	0.044
Vascular invasion (yes) (%)	276 (27.9%)	161 (33.5%)	115 (22.5%)	<0.001
AFP (ng/ml)	59.60; 10.10–1056.50	89.20; 13.50–1490.00	40.15; 7.78–741.95	0.864
**Tumor staging and treatment modality**				
BCLC stage (0/A/B/C/D)	65/277/274/295/79 (6.6%/28.0%/27.7%/29.8%/8.0%)	33/126/100/160/61 (6.9%/26.3%/20.8%/ 33.3%/12.7%)	32/151/174/135/18 (6.3%/29.6%/34.1% /26.5%/3.5%)	<0.001
Treatment modality (Resection surgery/RFA/TACE/others^$^)	179/195/287/329 (18.1%/19.7%/29.0%/33.2%)	31/103/134/212 (6.5%/21.5%/27.8%/44.2%)	148/92/153/117 (29.0%/18.0%/30.0%/23.0%)	<0.001
Treatment modality (curative/non-curative)	386/604 (39.0%/61.0%)	143/337 (29.8%/70.2%)	243/267 (47.6%/52.4%)	<0.001

^$^Others: best supportive therapy: 196, chemotherapy: 7, sorafenib: 79, radiotherapy: 34, chemo-radiotherapy combination: 1, liver transplantation: 12.

The continuous variables are expressed as median; *IQR*.

Abbreviations: EGV; esophagogastric varices; HBsAg: hepatitis B surface antigen; HCV: hepatitis C virus; MELD: model for end-stage liver disease; ALT: alanine aminotransferase; AST: aspartate aminotransferase; Alk-P: alkaline phosphate; PT INR: prothrombin time international normalized ratio; AFP: alpha-fetoprotein; BCLC: the Barcelona Clinic Liver Cancer; TACE: transarterial chemoembolization.

**Table 2:** Factors associated with poor overall survival in HCC.

Variable	Case No.	Univariate analysis		Multivariate analysis	
		Hazard ratio (95% CI)	*p*	Hazard ratio (95% CI)	*p*
Age > 65/≤65 years	544/446	0.900(0.759–1.067)	0.225		
Sex male/female	756/234	1.407(1.140–1.737)	0.001	1.425(1.146–1.773)	0.001
HBsAg positive/negative	536/452	1.233(1.039–1.464)	0.017		
Anti-HCV positive/negative	305/684	0.814(0.676–0.981)	0.031		
Albumin ≤4/>4 g/dL	778/209	2.182(1.717–2.774)	<0.001	1.621(1.259–2.088)	<0.001
Bilirubin >1.6/≤1.6 mg/dL	235/755	2.191(1.821–2.636)	<0.001	1.702(1.390–2.085)	<0.001
ALT > 40/≤40 U/L	583/407	1.330(1.115–1.585)	0.002		
AST > 45/≤45U/L	657/319	2.268(1.852–2.778)	<0.001	1.257(1.008–1.567)	0.043
Platelet ≤10^5^/>10^5^/mm^3^	329/661	1.224(1.023–1.464)	0.027	1.247(1.030–1.511)	0.024
PT INR > 1.1/≤1.1	489/501	1.424(1.200–1.690)	<0.001		
Ascites (yes/no)	307/683	3.108(2.613–3.696)	<0.001		
AFP > 20/≤20 ng/ml	636/353	2.553(2.092–3.116)	<0.001	1.863(1.504–2.307)	<0.001
Multiple tumor (yes/no)	447/543	1.823(1.536–2.163)	<0.001	1.233(1.023–1.488)	0.028
Tumor size >3/≤3 cm	669/321	3.292(2.656–4.081)	<0.001	2.346(1.838–2.996)	<0.001
Vascular invasion (yes/no)	275/715	4.827(4.018–5.800)	<0.001	2.600(2.124–3.183)	<0.001
Treatment modality (non-curative/curative)	604/386	4.029(3.291–4.932)	<0.001	1.884(1.485–2.392)	<0.001
EGV (yes/no)	480/510	1.792(1.509–2.129)	<0.001	1.324(1.099–1.596)	0.003

Abbreviations: CI: confidence interval; HBsAg: hepatitis B surface antigen; HCV: hepatitis C virus; MELD: model for end-stage liver disease; ALT: alanine aminotransferase; AST: aspartate aminotransferase; Alk-P: alkaline phosphate; PT INR: prothrombin time international normalized ratio; AFP: alpha-fetoprotein; EGV: esophagogastric varices.

**Table 3:** Poor prognostic factors of HCC after curative therapy.

Variable	Case No.	Univariate analysis		Multivariate analysis	
		Hazard ratio (95% CI)	*p*	Hazard ratio (95% CI)	*p*
Age >65/≤65 years	215/171	1.297(0.909–1.850)	0.151		
Sex male/female	280/106	1.116(0.752–1.658)	0.585		
HBsAg positive/negative	207/179	0.905(0.641–1.277)	0.569		
Anti-HCV positive/negative	134/252	0.979(0.683–1.403)	0.908		
Albumin ≤4/>4 g/dL	258/126	1.698(1.134–2.544)	0.010		
Bilirubin >1.6/≤1.6 mg/dL	50/336	1.755(1.126–2.735)	0.013		
ALT > 40/≤40 U/L	210/176	1.249(0.881–1.771)	0.211		
AST > 45/≤45 U/L	210/175	1.740(1.218–2.485)	0.002		
Platelet ≤10^5^/>10^5^/mm^3^	125/261	1.990(1.403–2.823)	<0.001	1.909(1.303–2.797)	0.001
PT INR > 1.1/≤1.1	159/227	1.437(1.012–2.041)	0.043		
Ascites (yes/no)	63/323	1.938(1.305–2.877)	0.001		
AFP > 20/≤20 ng/ml	209/177	2.096(1.456–3.016)	<0.001	1.674(1.144–2.450)	0.008
Multiple tumor (yes/no)	94/292	1.398(0.960–2.034)	0.080		
Tumor size >3/≤3 cm	166/220	1.532(1.086–2.161)	0.015	1.844(1.249–2.723)	0.002
Vascular invasion (yes/no)	21/365	3.659(2.009–6.665)	<0.001	3.312(1.797–6.103)	<0.001
EGV (yes/no)	143/243	1.597(1.129–2.257)	0.008	1.629(1.117–2.376)	0.011

Abbreviations: CI: confidence interval; HBsAg: hepatitis B surface antigen; HCV: hepatitis C virus; MELD: model for end-stage liver disease; ALT: alanine aminotransferase; AST: aspartate aminotransferase; Alk-P: alkaline phosphate; PT INR: prothrombin time international normalized ratio; AFP: alpha-fetoprotein; EGV: esophagogastric varices.

**Table 4:** Poor prognostic factors of HCC after non-curative therapy.

Variable	Case No.	Univariate analysis		Multivariate analysis	
		Hazard ratio (95% CI)	*p*	Hazard ratio (95% CI)	*p*
Age > 65/≤65 years	329/275	0.758(0.623–0.922)	0.005		
Sex male/female	476/128	1.459(1.136–1.874)	0.003	1.487(1.150–1.924)	0.003
HBsAg positive/negative	329/273	1.510(1.238–1.843)	<0.001		
Anti-HCV positive/negative	171/432	0.804(0.645–1.000)	0.050		
Albumin ≤4/>4 g/dL	520/83	1.776(1.312–2.405)	<0.001	1.773(1.293–2.432)	<0.001
Bilirubin >1.6/≤1.6 mg/dL	185/419	1.799(1.464–2.210)	<0.001	1.875(1.508–2.332)	<0.001
ALT > 40/≤40 U/L	373/231	1.227(1.001–1.505)	0.049		
AST > 45/≤45 U/L	447/144	1.872(1.455–2.409)	<0.001		
Platelet ≤10^5^/>10^5^/mm^3^	191/413	1.025(0.831–1.265)	0.818		
PT INR > 1.1/≤1.1	330/274	1.206(0.990–1.468)	0.063		
Ascites (yes/no)	244/360	2.851(2.334–3.482)	<0.001		
AFP > 20/≤20 ng/ml	427/176	2.360(1.855–3.003)	<0.001	1.975(1.532–2.546)	<0.001
Multiple tumor (yes/no)	353/251	1.178(0.965–1.439)	0.108		
Tumor size > 3/≤3 cm	503/101	3.112(2.242–4.318)	<0.001	2.615(1.855–3.688)	<0.001
Vascular invasion (yes/no)	254/350	3.076(2.510–3.771)	<0.001	2.458(1.978–3.054)	<0.001
EGV (yes/no)	337/267	1.492(1.221–1.822)	<0.001	1.254(1.013–1.553)	0.038

Abbreviations: CI: confidence interval; HBsAg: hepatitis B surface antigen; HCV: hepatitis C virus; MELD: model for end-stage liver disease; ALT: alanine aminotransferase; AST: aspartate aminotransferase; Alk-P: alkaline phosphate; PT INR: prothrombin time international normalized ratio; AFP: alpha-fetoprotein; EGV: esophagogastric varices.

## References

[b1] El-Serag H. B. Epidemiology of viral hepatitis and hepatocellular carcinoma. Gastroenterology 142, 1264–1273; doi: 10.1053/j.gastro.2011.12.061 (2012). 22537432 PMC3338949

[b2] Siegel R. L., Miller K. D. & Jemal A. Cancer statistics, 2015. CA Cancer J Clin 65, 5–29; doi: 10.3322/caac.21254 (2015). 25559415

[b3] Santi V. . The changing scenario of hepatocellular carcinoma over the last two decades in Italy. J Hepatol 56, 397–405; doi: 10.1016/j.jhep.2011.05.026 (2012). 21756850

[b4] Altekruse S. F., McGlynn K. A., Dickie L. A. & Kleiner D. E. Hepatocellular carcinoma confirmation, treatment, and survival in surveillance, epidemiology, and end results registries, 1992–2008. Hepatology 55, 476–482; doi: 10.1002/hep.24710 (2012). 21953588 PMC3868012

[b5] Kudo M. Surveillance, diagnosis, treatment, and outcome of liver cancer in Japan. Liver Cancer 4, 39–50; doi: 10.1159/000367727 (2015). 26020028 PMC4439792

[b6] Yim S. Y. . The management and prognosis of patients with hepatocellular carcinoma: what has changed in 20 years? Liver Int 36, 445–453; doi: 10.1111/liv.12960 (2016). 26352789

[b7] Nault J. C. . A hepatocellular carcinoma 5-gene score associated with survival of patients after liver resection. Gastroenterology 145, 176–187; doi: 10.1053/j.gastro.2013.03.051 (2013). 23567350

[b8] Su C. W. . Impact of steatosis on prognosis of patients with early-stage hepatocellular carcinoma after hepatic resection. Ann Surg Oncol 22, 2253–2261; doi: 10.1245/s10434-014-4221-5 (2015). 25490872

[b9] Berzigotti A., Reig M., Abraldes J. G., Bosch J. & Bruix J. Portal hypertension and the outcome of surgery for hepatocellular carcinoma in compensated cirrhosis: a systematic review and meta-analysis. Hepatology 61, 526–536; doi: 10.1002/hep.27431 (2015). 25212123

[b10] Su C. W. . The influence of hepatitis B viral load and pre-S deletion mutations on post-operative recurrence of hepatocellular carcinoma and the tertiary preventive effects by anti-viral therapy. PLoS One 8, e66457; doi: 10.1371/journal.pone.0066457 (2013). 23805222 PMC3689837

[b11] Hsu C. Y. . Performance status in patients with hepatocellular carcinoma: determinants, prognostic impact, and ability to improve the Barcelona Clinic Liver Cancer system. Hepatology 57, 112–119; doi: 10.1002/hep.25950 (2013). 22806819

[b12] D’Avola D. . Prognosis of hepatocellular carcinoma in relation to treatment across BCLC stages. Ann Surg Oncol 18, 1964–1971; doi: 10.1245/s10434-011-1551-4 (2011). 21267791

[b13] Walker M. . Cirrhosis is under-recognised in patients subsequently diagnosed with hepatocellular cancer. Aliment Pharmacol Ther 43, 621–630; doi: 10.1111/apt.13505 (2016). 26784271 PMC4742403

[b14] Chen P. H. . Delayed endoscopy increases re-bleeding and mortality in patients with hematemesis and active esophageal variceal bleeding: a cohort study. J Hepatol 57, 1207–1213; doi: 10.1016/j.jhep.2012.07.038 (2012). 22885718

[b15] Reverter E. . A MELD-based model to determine risk of mortality among patients with acute variceal bleeding. Gastroenterology 146, 412–419; doi: 10.1053/j.gastro.2013.10.018 (2014). 24148622

[b16] de Franchis R. & Baveno V. I. F. Expanding consensus in portal hypertension: Report of the Baveno VI consensus workshop: stratifying risk and individualizing care for portal hypertension. J Hepatol 63, 743–752; doi: 10.1016/j.jhep.2015.05.022 (2015). 26047908

[b17] Garcia-Tsao G. & Bosch J. Varices and variceal hemorrhage in cirrhosis: A new view of an old problem. Clin Gastroenterol Hepatol 13, 2109–2117; doi: 10.1016/j.cgh.2015.07.012 (2015). 26192141 PMC4851858

[b18] Giannini E. G. . Prevalence and prognostic significance of the presence of esophageal varices in patients with hepatocellular carcinoma. Clin Gastroenterol Hepatol 4, 1378–1384; doi: 10.1016/j.cgh.2006.08.011 (2006). 17059899

[b19] Bucci L. . Comparison between alcohol- and hepatitis C virus-related hepatocellular carcinoma: clinical presentation, treatment and outcome. Aliment Pharmacol Ther 43, 385–399; doi: 10.1111/apt.13485 (2016). 26662476

[b20] Harada N. . Surgical resection for hepatocellular carcinoma with concomitant esophageal varices. World J Surg 39, 2510–2518; doi: 10.1007/s00268-015-3110-9 (2015). 26059406

[b21] Ishizawa T. . Neither multiple tumors nor portal hypertension are surgical contraindications for hepatocellular carcinoma. Gastroenterology 134, 1908–1916; doi: 10.1053/j.gastro.2008.02.091 (2008). 18549877

[b22] Merkel C. & Montagnese S. Hepatic venous pressure gradient measurement in clinical hepatology. Dig Liver Dis 43, 762–767; doi: 10.1016/j.dld.2011.03.002 (2011). 21549649

[b23] D’Amico G. . Competing risks and prognostic stages of cirrhosis: a 25-year inception cohort study of 494 patients. Aliment Pharmacol Ther 39, 1180–1193; doi: 10.1111/apt.12721 (2014). 24654740

[b24] Bruix J. & Sherman M. Management of hepatocellular carcinoma: an update. Hepatology 53, 1020–1022; doi: 10.1002/hep.24199 (2011). 21374666 PMC3084991

[b25] Berzigotti A. . Elastography, spleen size, and platelet count identify portal hypertension in patients with compensated cirrhosis. Gastroenterology 144, 102–111; doi: 10.1053/j.gastro.2012.10.001 (2013). 23058320

[b26] Qamar A. A. . Platelet count is not a predictor of the presence or development of gastroesophageal varices in cirrhosis. Hepatology 47, 153–159; doi: 10.1002/hep.21941 (2008). 18161700

[b27] Groszmann R. J. . Beta-blockers to prevent gastroesophageal varices in patients with cirrhosis. N Engl J Med 353, 2254–2261; doi: 10.1056/NEJMoa044456 (2005). 16306522

[b28] Tsochatzis E. A., Bosch J. & Burroughs A. K. Liver cirrhosis. Lancet 383, 1749–1761; doi: 10.1016/S0140-6736(14)60121-5 (2014). 24480518

[b29] Cucchetti A. . Hepatic venous pressure gradient in the preoperative assessment of patients with resectable hepatocellular carcinoma. J Hepatol 64, 79–86; doi: 10.1016/j.jhep.2015.08.025 (2016). 26325538

[b30] Bruix J., Reig M. & Sherman M. Evidence-based diagnosis, staging, and treatment of patients with hepatocellular carcinoma. Gastroenterology 150, 835–853; doi: 10.1053/j.gastro.2015.12.041 (2016). 26795574

[b31] European Association for the Study of the Liver, European Organisation for Research and Treatment of Cancer. EASL-EORTC clinical practice guidelines: management of hepatocellular carcinoma. J Hepatol 56, 908–943; doi: 10.1016/j.jhep.2011.12.001 (2012). 22424438

[b32] Llovet J. M., Fuster J. & Bruix J. Intention-to-treat analysis of surgical treatment for early hepatocellular carcinoma: resection versus transplantation. Hepatology 30, 1434–1440; doi: 10.1002/hep.510300629 (1999). 10573522

[b33] Bruix J. . Surgical resection of hepatocellular carcinoma in cirrhotic patients: prognostic value of preoperative portal pressure. Gastroenterology 111, 1018–1022 (1996). 8831597 10.1016/s0016-5085(96)70070-7

[b34] Cucchetti A. . Is portal hypertension a contraindication to hepatic resection? Ann Surg 250, 922–928; doi: 10.1097/SLA.0b013e3181b977a5 (2009). 19855258

[b35] Roayaie S. . The role of hepatic resection in the treatment of hepatocellular cancer. Hepatology 62, 440–451; doi: 10.1002/hep.27745 (2015). 25678263

[b36] Santambrogio R. . Hepatic resection for hepatocellular carcinoma in patients with Child-Pugh’s A cirrhosis: is clinical evidence of portal hypertension a contraindication? HPB (Oxford) 15, 78–84; doi: 10.1111/j.1477-2574.2012.00594.x (2013). 23216782 PMC3533715

[b37] Hung H. H. . Fibrosis and AST to platelet ratio index predict post-operative prognosis for solitary small hepatitis B-related hepatocellular carcinoma. Hepatol Int 4, 691–699; doi: 10.1007/s12072-010-9213-3 (2010). 21286339 PMC2994617

[b38] Arguedas M. R., McGuire B. M., Fallon M. B. & Abrams G. A. The use of screening and preventive therapies for gastroesophageal varices in patients referred for evaluation of orthotopic liver transplantation. Am J Gastroenterol 96, 833–837; doi: 10.1111/j.1572-0241.2001.03627.x (2001). 11280560

[b39] Matsunaga K. . Endoscopic injection sclerotherapy for esophageal varices in cirrhotic patients with hepatocellular carcinoma: risk factors for survival. J Clin Gastroenterol 36, 68–71 (2003). 12488712 10.1097/00004836-200301000-00018

[b40] Kim J. H. . Primary prophylaxis for variceal bleeding and the improved survival of patients with newly diagnosed hepatocellular carcinoma. Dig Dis Sci 61, 3354–3362; doi: 10.1007/s10620-016-4255-6 (2016). 27435325

[b41] Hung H. H. . Survival rates are comparable after radiofrequency ablation or surgery in patients with small hepatocellular carcinomas. Clin Gastroenterol Hepatol 9, 79–86; doi: 10.1016/j.cgh.2010.08.018. 20831902

[b42] Chen P. H. . Comparison of prognosis by viral etiology in patients with hepatocellular carcinoma after radiofrequency ablation. Ann Hepatol 12, 263–273 (2013). 23396738

