# Supplementary material for: The impact of esophagogastric varices on the prognosis of patients with hepatocellular carcinoma
Source: Sci Rep. 2017 Feb 17;7:42577. doi: 10.1038/srep42577 (PMC5314332; doi:10.1038/srep42577)
Supplement: Supplementary Table S1 [file srep42577-s1.doc]

**Supplementary information**

**Title of manuscript: The impact of esophagogastric varices on the prognosis of patients with hepatocellular carcinoma**

**Short title:** Esophagogastric varices and HCC prognosis

**Author list:** Wei-Yao Hsieh, MD; Ping-Hsien Chen, MD; I-Yen Lin, BS;

Chien-Wei Su, MD & PhD; Yee-Chao, MD; Teh-Ia Huo, MD;

Yi-Hsiang Huang, MD & PhD; Ming-Chih Hou, MD; Han-Chieh Lin, MD; Jaw-Ching Wu, MD & PhD

**Supplementary Table S1. Comparison of the demographic data of HCC patients with and without EGV by propensity analysis with one-to-one nearest-neighbor matching method**

| **Parameter** | | **All patients**  **(n=354)** | | **EGV group**  **(n=177)** | **Non-EGV group**  **(n=177)** | ***P*** |
| --- | --- | --- | --- | --- | --- | --- |
| **Patient Demographics** | | | | | | |
| Age (years) | | 67.0; 57.8-78.0 | | 67.0; 57.0-76.5 | 68.0; 58.0-79.0 | 0.393 |
| Sex (male) (%) | | 281 (79.4%) | | 141 (79.7%) | 140 (79.1%) | 1.000 |
| HBsAg positive/negative (%) | | 194/159  (55.0%/45.0%) | | 95/82  (53.7%/46.3%) | 99/77  (56.3%/43.7%) | 0.704 |
| Anti-HCV positive/negative (%) | | 106/247  (30.0%/70.0%) | | 54/123  (30.5%/69.5%) | 52/124  (29.5%/70.5%) | 0.935 |
| MELD score | | 8.81;7.60-11.37 | | 8.98;7.80-11.76 | 8.44;7.34-11.31 | 0.835 |
| **Serum biochemistry tests** | |  | |  |  |  |
| Albumin (g/dL) | | 3.50; 3.10-3.90 | | 3.50; 3.10-3.85 | 3.60; 3.15-4.00 | 0.330 |
| Total bilirubin (mg/dL) | | 0.93; 0.63-1.54 | | 1.03; 0.69-1.65 | 0.82; 0.60-1.31 | 0.555 |
| ALT (U/L) | | 49.0; 31.8-78.5 | | 49.0; 31.5-83.0 | 49.0; 31.5-75.5 | 0.876 |
| AST (U/L) | | 67.5; 40.8-110.5 | | 69.0; 44.0-113.5 | 67.0; 38.5-103.0 | 1.000 |
| Alk-P (U/L) | | 114.5; 83.8-161.3 | | 116.0; 92.0-161.0 | 107.0; 81.0-164.0 | 0.764 |
| Cholesterol (U/L) | | 153.5; 126.8-173.5 | | 153.5; 123.3-183.5 | 153.5; 128.0-172.3 | 0.731 |
| Creatinine (mg/dL) | | 0.88; 0.73-1.10 | | 0.86; 0.71-1.08 | 0.90; 0.75-1.14 | 0.507 |
| Glucose (mg/dL) | | 97.5; 83.0-129.3 | | 101.0; 82.5-134.0 | 97.0; 83.5-125.0 | 0.806 |
| PT INR | | 1.10; 1.04-1.18 | | 1.11; 1.06-1.20 | 1.09; 1.03-1.17 | 0.475 |
| Platelet (/mm^3^) | | 127000;  83000-179250 | | 127000;  80500-187500 | 127000;  85500-174000 | 0.627 |
| Ascites (yes) (%) | | 102 (28.8%) | | 54 (30.5%) | 48 (27.1%) | 0.557 |
| Hepatic encephalopathy (yes/no) | | 8/265 (2.9%/97.1%) | | 4/133 (2.9%/97.1%) | 4/132 (2.9%/97.1%) | 1.000 |
| Hemoglobin (g/dL) | | 12.2;10.4-13.8 | | 12.1;10.3-13.8 | 12.3;10.6-14.0 | 0.173 |
| Child-Pugh grade (A/B/C) (%) | | 244/94/16  (68.9%/26.6%/4.5%) | | 117/53/7  (66.1%/29.9%/4.0%) | 127/41/9  (71.8%/23.2%/5.0%) | 0.334 |
| **Tumor factors** | |  | |  |  |  |
| Tumor size (cm) | | 5.60; 2.68-10.00 | | 5.20; 2.60-10.00 | 6.00; 3.00-10.00 | 0.265 |
| Single tumor (%) | | 192 (54.2%) | | 101 (57.1%) | 91 (51.4%) | 0.337 |
| Vascular invasion (yes) (%) | | 104 (29.4%) | | 57 (32.2%) | 47 (26.6%) | 0.294 |
| AFP (ng/ml) | | 90.75; 14.48-1315.00 | | 101.00; 15.95-1860.95 | 54.80;11.80-1010.00 | 0.416 |
| **Tumor staging and treatment modality** | | | | | | |
| BCLC stage (0/A/B/C/D) | 31/96/95/109/23 (8.8%/27.1%/26.8%  /30.8%/6.5%) | | 16/50/42/59/10 (9.0%/28.3%/23.7%/  33.3%/5.7%) | | 15/46/53/50/13 (8.5%/26.0%/29.9%  /28.3%/7.3%) | 0.626 |
| Treatment modality  (curative/non-curative) | 137/217(38.7%/61.3%) | | 69/108(39.0%/61.0%) | | 68/109(38.4%/61.6%) | 1.000 |
